# Inhibitory Mechanisms of Vine Tea Extract and Dihydromyricetin Against *Escherichia coli*: A Multidimensional Analysis from Cell Membrane to Protein Synthesis

**DOI:** 10.3390/foods14122011

**Published:** 2025-06-06

**Authors:** Wei Ma, Haiyun Liang, Keke He, Ting Li, Qiaoni Hui, Yao Zhang, Yuxuan Dong, Yan Jia, Liya Song

**Affiliations:** Department of Cosmetics, School of Light Industry Science and Engineering, Beijing Technology and Business University, Beijing 100048, China; mawei901@163.com (W.M.); 15216856275@163.com (H.L.); 13121516520@163.com (K.H.); qianandeliting@126.com (T.L.); huiqiaoni1111@163.com (Q.H.); jiajingwenzhangyao@126.com (Y.Z.); socializedyx@163.com (Y.D.)

**Keywords:** vine tea extract, dihydromyricetin, *Escherichia coli*, proteomics, bacterial lipids, molecular docking

## Abstract

Microbial contamination is the leading cause of foodborne diseases and spoilage in food and personal care products. Previous studies by our group have demonstrated that vine tea extract (VTE) and dihydromyricetin (DMY) inhibit the growth of *Escherichia coli*. In this study, we further explored the inhibitory mechanisms of VTE and DMY against *E. coli* through a label-free proteomics approach. The proteomic analysis detected 130 and 81 differentially expressed proteins (DEPs) in *E.coli* following VTE and DMY treatment, respectively. The analysis indicated that VTE and DMY inhibit bacterial growth through multiple-target mechanisms. Specifically, they inhibit *E. coli* growth by disrupting the cationic antimicrobial peptide resistance pathway, amino acid biosynthesis and metabolism, and nucleotide metabolism. Additionally, VTE disrupts various secondary metabolic pathways, while DMY interferes with *E. coli* ribosome assembly and function, and disrupts cell membrane lipid homeostasis by interfering with fatty acid metabolism. RT-qPCR validation confirmed transcriptional alterations in genes encoding key target proteins. Molecular docking results indicated that DMY may affect bacterial protein synthesis, cationic antimicrobial peptide resistance, and transcriptional regulation by binding to target proteins such as RplB, RplV, LpxA, and YafC. In conclusion, this study systematically deciphered the multi-target inhibitory mechanisms of VTE and DMY against *E. coli*, providing a theoretical basis for developing plant-derived antimicrobial agents.

## 1. Introduction

*Escherichia coli* is a Gram-negative bacterium commonly found in the intestinal tract of humans and animals; certain strains are pathogenic. It is an aerobic or facultatively anaerobic bacterium, known for its high adaptability to various environments. *E. coli* is a leading cause of foodborne illnesses globally, particularly in contaminated raw foods, water, vegetables, and meat [1]. Certain pathogenic strains, such as *E. coli* O157:H7, produce Shiga toxin, which can cause severe foodborne illnesses. Symptoms include diarrhea, vomiting, abdominal pain, and, in severe cases, hemolytic uremic syndrome, representing a significant public health threat [2,3]. Although antibiotics effectively treat *E. coli* infections in modern medicine, their overuse and misuse—particularly as growth promoters in animal husbandry—have contributed to increased antibiotic resistance in *E. coli* strains [4]. Consequently, the development of new antimicrobial agents to control the spread and infection of *E. coli* has become a pressing global concern.

Vine tea is derived from the young stems and leaves of *Ampelopsis grossedentata*, a plant belonging to the genus *Ampelopsis*, and is a traditional medicinal and food plant commonly found in southern China. It has been used for centuries as both a health tea and an herbal medicine. Vine tea contains active compounds such as polyphenols, flavonoids, and fatty acids [5]. Dihydromyricetin (DMY) is the most abundant flavonoid in vine tea. Studies have shown that dihydromyricetin exhibits antioxidant [6], anti-inflammatory [7], antimicrobial [8], anticancer [9,10], anti-obesity [11], and immunomodulatory [12] activities. Currently, DMY has been shown to inhibit *Staphylococcus aureus*, *Pseudomonas aeruginosa*, *Bacillus subtilis*, and *Vibrio parahaemolyticus* [13,14]. Previous studies from our group have demonstrated that VTE and DMY inhibit bacterial growth by affecting the integrity and fluidity of the bacterial cell wall membranes and by directly interacting with *E. coli* DNA [15].

Therefore, understanding the protein-level response of *E. coli* to VTE and DMY will provide a more comprehensive understanding of their antimicrobial mechanisms. Proteomics is an effective tool for studying changes in bacterial proteins induced by external stressors and has been widely used to investigate bacterial inhibition mechanisms. Lipidomics is an emerging field that uses a systems-based approach to study lipids, their interacting molecules, and their functions within the cell [16]. In this study, we applied proteomics to identify differentially expressed proteins (DEPs) in *E. coli* treated with VTE and DMY. Additionally, lipid assays, RT-qPCR, and molecular docking were employed to further elucidate the antibacterial mechanisms of VTE and DMY against *E. coli* in detail. This study provides a theoretical foundation for the use of VTE and DMY as novel preservatives in food and daily chemicals.

## 2. Materials and Methods

### 2.1. Materials and Reagents

*E. coli* was sourced from the China Medical Culture Collection Center (Beijing, China). In May 2019, newly germinated spear leaves were collected from Zhangjiajie, Hunan Province. Dried and crushed vine tea (10 g) was extracted with 100 mL of deionized water at 100 °C for 1 h. The combined filtrates were concentrated to 20 mL using a rotary evaporator and stored at 4 °C for future experiments. DMY was obtained from Beijing Banxia Biotechnology Co. located in Beijing, China. All other chemical reagents, including Na_2_CO_3_, anhydrous ethanol, methanol, phenylmethylsulfonyl fluoride, iodoacetamide, and borane-triethylamine complex (Solarbio, Beijing, China) were of analytical grade. Nutrient broth and nutrient agar were purchased from Kainuo Spring Biotechnology Co. in Beijing, China.

### 2.2. Sample Preparation

*E. coli*, in the logarithmic growth phase, was inoculated into LB broth, resulting in a final bacterial suspension concentration of 1 × 10^7^ CFU/mL. A total of 1 MIC of VTE or DMY was added, and the sample was incubated for 24 h. The bacterial cells were then precipitated by centrifugation (14,000 rpm, 4 °C for 5 min), washed three times with PBS buffer (Thermo Fisher Scientific, Waltham, MA, USA), and freeze-dried under vacuum. A total of 1 mL of bacterial lysate (8 M urea, 62 mM DTT, 1% PMSF, and 2% m/v CHAPS) was added per 25 mg of dry cell weight and mixed with a vortex. The mixture was placed on ice and vortexed every 2 min for 10 s to ensure thorough shaking. The cells were then processed using a cell sonicator (Sainz Biotechnology Co., Ningbo, China) for 15 min, and the supernatant was collected by centrifugation. Protein concentration was determined using the Bradford method, and an aliquot containing equal amounts of protein was prepared for SDS-PAGE analysis.

### 2.3. Protein Mass Spectrometry

The protein samples were mixed with pre-cooled acetone (Thermo Fisher Scientific, Waltham, MA, USA) at a 1:4 volume ratio and incubated at −20 °C overnight. The samples were then centrifuged at 14,000 rpm for 10 min, and the supernatant was discarded. The precipitates were washed twice with pre-cooled acetone at −20 °C, and after the acetone was fully absorbed, the proteins were resuspended in triethylammonium bicarbonate buffer (Thermo Fisher Scientific, Waltham, MA, USA). Subsequently, dithiothreitol (Beyotime, Shanghai, China) was added to each sample to achieve a final concentration of 5 mM, and the reaction was carried out at 56 °C for 30 min. Iodoacetamide (Aladdin, Shanghai, China) was added to each sample to achieve a final concentration of 10 mM, and the reaction was carried out in the dark at room temperature for 30 min. Lys-C protease (Roche, Penzberg, Bavaria, Germany) was added at a mass ratio of 1:50 (enzyme–protein), and digestion was performed at 37 °C for 3 h. Trypsin (Aladdin, Shanghai, China) was added at a mass ratio of 1:50 (enzyme–protein), and digestion was carried out at 37 °C for 12 h. The digestion was terminated by adding trifluoroacetic acid (Aladdin, Shanghai, China) to the digest, resulting in a final concentration of 5%. The digest was then desalted using a Sep-Pak C18 column and concentrated under vacuum. The sample was then analyzed using a nano UPLC-Q-TOF-MS/MS mass spectrometer (Waters Corporation, Milford, MA, USA). MassPREPTM was used as a quantitative standard. Bacterial proteins were analyzed for differential substances using Progenesis QI for Proteomics (Waters Corporation, Milford, MA, USA), and the system was validated with the Waters proteomics system validation suite.

### 2.4. Determination of Lipid Content

Log-phase *E. coli* was incubated with 1MIC of DMY for 12 h, with untreated cells serving as a control. The cells were then collected at 4 °C, washed twice with PBS buffer (0.1 M, pH = 7.2), and lyophilized. A total of 50 mg of dried cell precipitates were suspended in an extraction solution (chloroform–methanol = 2:1, *v*/*v*) and homogenized using an ultrasonic cell pulverizer (Ningbo Saints Biotechnology Co., Ningbo, China) at a 3% intensity with 2-second intermittent pulses. The cell homogenate was centrifuged, and the supernatant was concentrated to 100 μL. Membrane lipid changes were analyzed using UPLC-Q-TOF-MS/MS spectroscopy. High-resolution mass measurements were conducted using a Waters Xevo G2-XSQTOF-MS (Waters Corporation, Milford, MA, USA) equipped with an electrospray ionization interface that operated in both positive and negative ion modes; detailed parameters are provided in Table A1. Data extraction and analysis were performed using Waters Progenesis QI 2.0 and Ezinfo 3.0 (Waters Corporation, Milford, MA, USA).

### 2.5. RNA Extraction and Quantification Real-Time Fluorescence Quantitative PCR

Bacterial samples were collected, frozen in liquid nitrogen, and subsequently ground. Total bacterial RNA was extracted using the Trizol method. cDNA was synthesized using the FastQuant RT Kit (with gDNase) (Tiangen Biotech, Beijing, China) and used as the template for RT-qPCR amplification with SuperReal PreMix Plus (SYBR Green) (Tiangen Biotech, Beijing, China) on the LightCycle 480 Real-Time PCR System (Roche, Basel, Switzerland). Primers were designed using Primer Premier 5.0 and are listed in Table A2.

### 2.6. Bioinformatics Analysis

The functions of the DEPs were analyzed using functional information from the Uniprot database (http://www.uniprot.org/,accessed on 5 August 2024). Gene Ontology (GO; http://geneontology.org/, accessed on 10 August 2024) was used for the functional clustering of all DEPs, focusing on biological processes, cellular components, and molecular functions for GO annotation analysis. The Kyoto Encyclopedia of Genes and Genomes (KEGG; http://www.genome.jp/kegg/, accessed on 3 September 2024) pathway database was used to analyze metabolic pathways associated with the DEPs. DEPs were analyzed, and protein–protein interaction networks were constructed using the online database STRING 12.0 (Search Tool for the Retrieval of Interacting Genes/Proteins; https://string-db.org/, accessed on 10 September 2024). The network was visualized using Cytoscape 3.8.2 (www.cytoscape.org, accessed on 1 December 2024), excluding orphan proteins (unlinked proteins) and satellite networks (networks disconnected from the main network).

### 2.7. Molecular Docking Analysis

Molecular docking studies were performed between DMY and key proteins from *E.coli*. Before docking, the protein was prepared with PyMol 3.1.1 to remove water molecules and ligands. Molecular docking was subsequently performed with AutoDock 4.2.6 software. Additionally, Open Babel was used for format conversion between PDB and PDBQT files. Finally, the docking results were visualized and analyzed with PyMol 3.1.1. The docking procedure was performed 50 times.

### 2.8. Statistical Analysis

All experiments were conducted in triplicate, and the results were presented as the mean ± standard deviation. Statistical analysis was conducted using GraphPad Prism 9 software. A *p*-value of less than 0.05 was considered statistically significant in all cases. Asterisks indicated statistically significant differences between groups (* *p* ≤ 0.05; ** *p* ≤ 0.01; *** *p* ≤ 0.001)

## 3. Results and Discussion

### 3.1. Identification of DEPs

**Figure 1:**
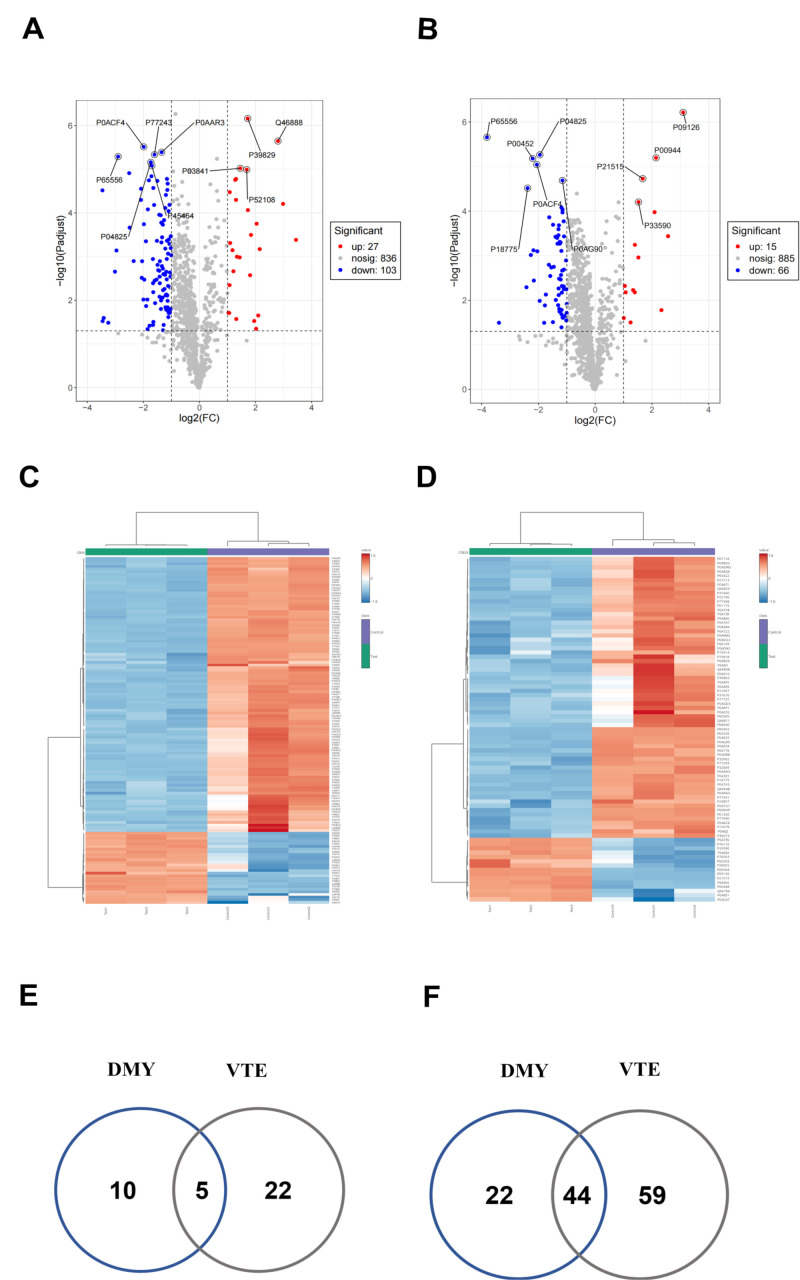
(**A**) DEP volcano map of VTE-treated *E.coli*. (**B**) DEP volcano map of DMY-treated *E.coli*. (**C**) Hierarchical clustering heatmap of DEPs in VTE-treated *E.coli*. (**D**) Hierarchical clustering heatmap of DEPs in DMY-treated *E.coli*. (**E**) DEPs jointly up-regulated in VTE-treated and DMY-treated groups. (**F**) DEPs jointly down-regulated in VTE-treated and DMY-treated groups.

A total of 1208 proteins were identified in the VTE-treated and control groups. Similarly, a total of 1183 proteins were identified in both the DMY-treated and control groups. Identified proteins were selected based on fold changes ≥2 or ≤0.5. A total of 130 DEPs were identified in the VTE-treated group compared to the control group. Among these, 27 proteins were significantly up-regulated, while 103 proteins were significantly down-regulated (Figure 1A). Furthermore, 81 DEPs were identified in the DMY-treated group compared to the control group, including 15 proteins that were significantly up-regulated and 66 proteins that were significantly down-regulated (Figure 1B). Hierarchical clustering heatmaps of DEPs in VTE- and DMY-treated *E. coli* are shown in Figure 1C,D, respectively. The Venn diagram also revealed that 5 DEPs were co-up-regulated in both the VTE-treated and DMY-treated groups (Figure 1E), while 44 DEPs were co-down-regulated (Figure 1F). Detailed data of DEPs are shown in Tables S1 and S2.

### 3.2. Functional Annotation and Pathway Enrichment

**Figure 2:**
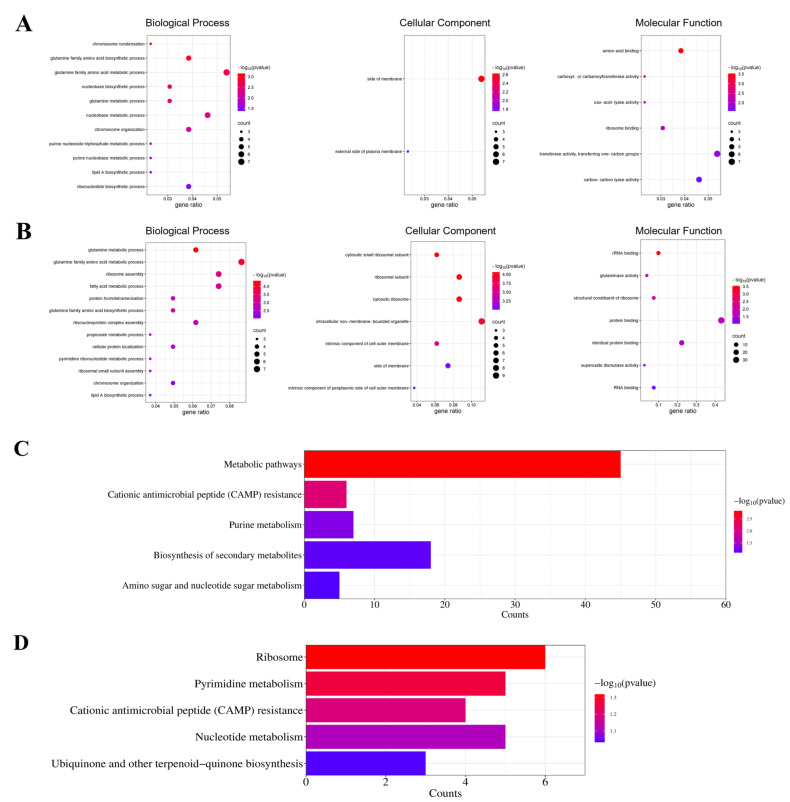
(**A**) GO enrichment analysis of DEPs in *E. coli* from VTE-treated groups. (**B**) GO enrichment analysis of DEPs in *E. coli* from DMY-treated groups. (**C**) KEGG enrichment analysis of DEPs of *E. coli* in VTE-treated group. (**D**) KEGG enrichment analysis of DEPs of *E. coli* in DMY-treated group.

GO enrichment analysis revealed that the DEPs in the VTE group were involved in biological processes (BP), including chromosome condensation, glutamine family amino acid biosynthesis, nucleobase metabolism, purine nucleoside triphosphate metabolism, and lipid A biosynthesis. The DEPs in the VTE group were associated with cellular components (CCs) located on the membrane side and the external side of the plasma membrane. In addition, the molecular functions (MFs) of the DEPs in the VTE group included ribosome binding, carbon–carbon lyase activity, oxo-acid-lyase activity, transferase activity, amino acid binding, and carboxyl- or carbamoyltransferase activity (Figure 2A). DEPs in the DMY group were involved in biological processes (BPs), including glutamine metabolism, ribosome assembly, fatty acid metabolism, protein homotetramerization, cellular protein localization, chromosome organization, and lipid A biosynthesis. The DEPs in the DMY group were associated with cellular components (CCs) located on the ribosomal subunit and intrinsic components of the cell outer membrane. Additionally, the molecular functions (MFs) of the DEPs in the DMY group included rRNA binding, glutaminase activity, protein binding, and superoxide dismutase activity (Figure 2B).

KEGG enrichment analysis indicated that the DEPs in the VTE group were enriched in pathways, including metabolic pathways, cationic antimicrobial peptide (CAMP) resistance, purine metabolism, the biosynthesis of secondary metabolites, and amino sugar and nucleotide sugar metabolism (Figure 2C). DEPs in the DMY group were enriched in pathways, including ribosomal pathways, pyrimidine metabolism, cationic antimicrobial peptide (CAMP) resistance, nucleotide metabolism, as well as ubiquinone and other terpenoid-quinone biosynthesis (Figure 2D).

### 3.3. Effects on E. coli Cell Membranes

#### 3.3.1. DEPs Associated with Cationic Antimicrobial Peptide Resistance

**Figure 3:**
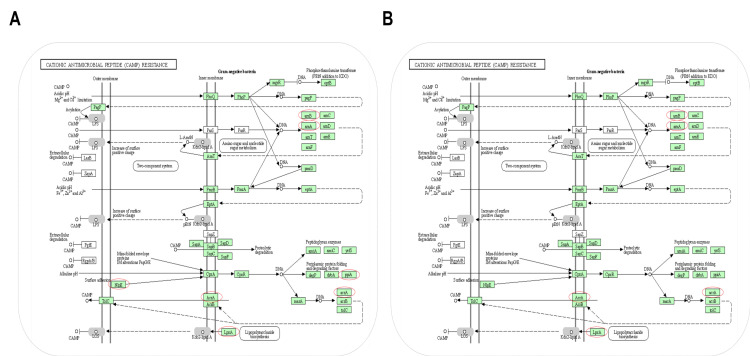
(**A**) Effect of VTE on cationic antimicrobial peptide resistance-related proteins in *E. coli*. (**B**) Effect of DMY on *E. coli* cationic antimicrobial peptide resistance-associated proteins. Red circles represent protein down-regulation.

Previous research by the group demonstrated that VTE and DMY influence the structural integrity and permeability of the *E. coli* cell wall and membrane. Enrichment analysis revealed that VTE and DMY affect the cationic antimicrobial peptide system in *E. coli*. Specifically, VTE and DMY significantly down-regulated six (ArnB, ArnA, AcrA, LpxA, PpiA, and NlpE) and four (ArnB, ArnA, AcrA, and LpxA) proteins, respectively, in this system (Figure 3). Experimental results showed that both VTE and DMY reduce LpxA expression in *E. coli*, thereby affecting lipid A synthesis. Gram-negative bacteria possess complex glycolipids called lipopolysaccharides (LPSs) on their cell surface, which mediate various interactions between the bacteria and their environment [17]. Lipid A is a phosphorylated glucosamine dimer substituted with fatty acyl chains, serving as the hydrophobic component of lipopolysaccharide (LPS), and is highly conserved. Lipid A, as part of the outermost defense layer of bacterial cells, plays a role in bacterial adaptation to the host environment and protection from harmful chemotaxis [18]. The Raetz pathway is one of the lipid A biosynthesis routes, synthesizing 3-deoxy-d-manno-octo-2-ulosonic acid 2 through a nine-step process, forming the lipid A substructure of *E. coli* LPS. The first step, catalyzed by LpxA, involves adding an acyl chain to the 3-OH group of uridine diphosphate N-acetylglucosamine [19]. Additionally, both VTE and DMY reduced the expression of ArnA and ArnB; VTE resulted in a 0.76-fold and 0.57-fold decrease in ArnA and ArnB expression, respectively, while DMY led to a 0.79-fold and 0.64-fold decrease in ArnA and ArnB expression. ArnA and ArnB are essential enzymes in the biosynthesis of ArnT, a transferase that catalyzes the addition of a 4-amino-4-deoxy-L-arabinose (4-Ara4N) group to the L-Ara4N moiety, which is covalently attached to the Lipid A portion of LPS [20]. The attachment of L-Ara4N to Lipid A is one mechanism by which Gram-negative bacteria, such as *E. coli*, maintain resistance to CAMPs [21]. The down-regulation of these proteins by VTE and DMY may affect cationic antimicrobial peptide resistance in *E. coli* through lipid A.

The cell envelope in Gram-negative bacteria consists of an inner membrane (IM) and an outer membrane (OM). To ensure survival in fluctuating environments, bacteria have several signaling systems known as envelope stress response systems (ESRSs), which monitor envelope biogenesis and homeostasis. The Cpx two-component system, an ESRS in *E. coli*, plays a protective role under various stresses, such as acidic alkalinity and high osmotic pressure [22]. The lipoprotein NlpE is a sensor of the Cpx envelope stress response and activates the Cpx signaling pathway during stress. Its envelope localization is sensitive to lipoprotein defects. Defects in acetylation, lipoprotein transport, or exposure to chemical inhibitors lead to the mislocalization of NlpE to the IM. Mislocalized NlpE triggers the activation of the Cpx pathway [23]. The subsequent Cpx response is crucial for *E. coli* survival under lipoprotein biogenesis stress. Meanwhile, the CpxA/CpxR two-component signaling system regulates the genes encoding cell envelope proteins involved in protein folding and degradation. Phosphorylated CpxR binds to the promoter region upstream of the PpiA transcription start site, enhancing in vivo PpiA transcription upon the activation of the CpxR pathway [24]. VTE treatment of *E. coli* reduces NlpE expression, which in turn decreases the activity of the CpxA-CpxR system and the expression of the PpiA protein. PpiA, a periplasmic cis–trans prolyl isomerase, promotes proper protein folding by accelerating the transition of proline residues between cis and trans states, whereas the genetic inactivation of this isomerase results in live strains with reduced growth rates and increased susceptibility to certain antibiotics [25]. Additionally, numerous proteins in the *E. coli* OM contribute to the bacterial resistance response. AcrA-AcrB-TolC is a key multidrug efflux system and one of the most active ABC transporters [26]. AcrA, a periplasmic auxiliary protein, is an elongated subunit that spans the periplasmic space and coordinates the synergistic action of the inner membrane transporter AcrB and the outer membrane protein TolC, ultimately pumping toxic molecules out of the IM through membrane pores [27]. The down-regulation of AcrA expression after VTE and DMY treatment suggests that these compounds reduce *E. coli* resistance to external toxins by affecting the AcrA-AcrB-TolC efflux system. In conclusion, VTE and DMY likely disrupt the bacterial membrane structure and function by targeting the cationic peptide resistance system, preventing *E. coli* from performing its normal resistance function in response to external stimuli.

#### 3.3.2. DEPs Associated with Fatty Acid Metabolism

Additionally, we found that DMY affects proteins involved in fatty acid metabolism in *E. coli*, including four down-regulated and two up-regulated proteins. In *E. coli*, several enzymes, including 2-methylisocitrate lyase (PrpB), 2-methylcitrate synthase (PrpC), and 2-methylcitrate dehydratase (PrpD), participate in the catabolism of short-chain fatty acids via the 2-methylcitrate cycle I, a pathway for propionate degradation [28]. The 2-methylcitrate cycle is a propionyl-CoA utilization pathway commonly found in bacteria. Impairment of the 2-methylcitric acid cycle, such as mutations or deletions of key enzyme genes, often leads to the accumulation of the cytotoxic intermediates 2-methylcitric acid and 2-methylisocitric acid [29]. Our results showed a down-regulation of PrpC, PrpD, and PrpB expression, suggesting that DMY affects the 2-methylcitrate cycle in *E. coli*, disrupting normal short-chain fatty acid metabolism and inducing cytotoxicity. Additionally, PlsB was down-regulated and AcpH was up-regulated following DMY treatment. Phospholipid biosynthesis begins with the acylation of glycerol-3-phosphate (G3P) to form 1-acyl-G3P, catalyzed by the PlsB protein in *E. coli* [30]. AcpH converts holo-ACP to apo-ACP by hydrolyzing phospholipid prosthetic groups from ACP and may play a role in bacterial lipid metabolism [31]. Additionally, LCFA metabolism can impede oxidative protein folding and activate envelope stress responses to restore bacterial homeostasis. One enzyme encoded by the fad regulator is involved in transport, acylation, and β-oxidation during LCFA metabolism [32]. Among these enzymes, 3-ketoacyl-CoA thiolase (FadA) catalyzes the final step of fatty acid oxidation, releasing acetyl-CoA and forming fatty acid-CoA esters two carbons shorter [33]. Studies show that FadI is a functional homolog of FadA with thiolase activity [34]. The up-regulation of FadI expression after DMY exposure suggests an impact on bacterial long-chain fatty acid metabolism. Our lipid analysis further confirmed the elevated LCFA content in *E. coli* after DMY treatment. Ubiquinone is a key antioxidant in the bacterial LCFA metabolic pathway, counteracting oxidative stress during LCFA degradation [35]. Proteomic results also indicated that the ubiquinone biosynthesis pathway was affected. These results suggested that DMY may disrupt normal fatty acid metabolism and membrane lipid homeostasis in *E. coli*, affecting both long- and short-chain fatty acid metabolism as well as phospholipid synthesis. DMY also impacts the antioxidant function of the bacterial cell, exacerbating oxidative stress in the bacterial envelope [36].

### 3.4. Lipidome Profiling Overview

**Figure 4:**
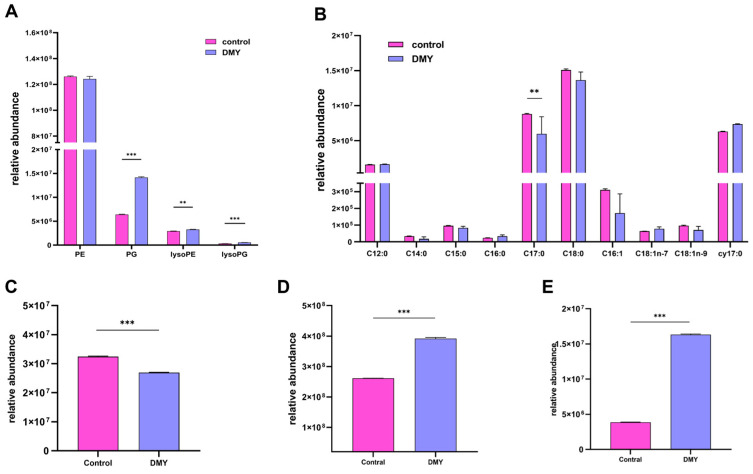
Effects of DMY on *E.coli* lipids: (**A**) Changes in membrane major lipids. (**B**) Changes in membrane fatty acids. (**C**) Changes in total fatty acids. (**D**) Changes in long-chain fatty acids. (**E**) Changes in short-chain fatty acids. **: *p* < 0.01, ***: *p* <0.001.

Based on the previous analysis, DMY was shown to impact fatty acid metabolism in *E. coli*. Consequently, we further investigated the effects of DMY on *E. coli* membrane lipids. Changes in lipid composition are a common cellular response to antimicrobial-induced stress. Amphipathic phosphatidylethanolamine (PE) and anionic phosphatidylglycerol (PG) are reported to constitute 75% and 20% of the total lipids in *E. coli* cells, respectively [37]. Additionally, trace amounts of anionic cardiolipin (CL), lysophosphatidylethanolamine (LysoPE), and lysophosphatidylglycerol (LysoPG) were detected [38]. Lipidomic analysis revealed that DMY treatment increased the lipid content of PG 2.2-fold, lysoPE 1.1-fold, and lysoPG 1.7-fold compared to untreated bacteria, while CL was not detected (Figure 4A). Notably, antimicrobial agents exert their antimicrobial activity by inducing lipid loss [39]; we observed an increase in lipid content in *E. coli* following DMY treatment. This increase may have resulted from the altered lipid composition, a typical bacterial response to antimicrobial-induced damage, whereby bacteria protect themselves by producing more membrane lipids [40].

In addition, the saturated fatty acids (C12:0, C14:0, C15:0, C16:0, C17:0, C18:0, cyclopropyl fatty acids cy17:0), as well as the unsaturated fatty acid C16:1 and two isomers of C18:1 (oleic acid C18:1N9, oxalic acid C18:1N11) found in *E. coli* membranes, were altered to varying degrees [41]. Specifically, 64.24%, 16.89%, 48.34%, 14.14%, 60.01%, and 41.24% of C14:0, C15:0, C17:0, C18:0, C16:1, and C18:1n−9 were lost, respectively (Figure 4B). Furthermore, the total fatty acid content decreased by 16.97% following DMY treatment (Figure 4C), while long-chain and short-chain fatty acids increased by 49.76% and 321.21%, respectively (Figure 4–E). Fatty acids are crucial components of bacterial cell membranes, and a reduction in their synthesis destabilizes the membrane structure [42]. The increased content of long-chain and short-chain fatty acids further corroborates the proteomics results. DMY may disrupt the normal metabolism of these fatty acids in *E. coli*, thereby disturbing the membrane’s fatty acid balance and compromising membrane structure and function.

### 3.5. Effects on E. coli Proteins

#### 3.5.1. DEPs Related to Amino Acid Biosynthesis and Metabolism

Glutamine is a key amino acid involved in amino acid synthesis [43], nitrogen supply [44], and cellular stress response [45]. Our results showed that exposure to VTE and DMY led to the down-regulation of CarA, AsnB, and HisH. CarA, the small subunit of glutamine-dependent carbamoylphosphate synthetase, binds and cleaves glutamine, providing ammonia as a substrate for the large subunit. CPSase catalyzes the formation of carbamoylphosphate from glutamine-derived ammonia, carbonate, and ATP-supplied phosphate, initiating two biosynthetic pathways. This step initiates two biosynthetic pathways: one leading to arginine or urea, and the other to pyrimidine nucleotides [46]. AsnB is a glutamine hydrolase that catalyzes the ATP-dependent conversion of aspartic acid to asparagine, using glutamine as a nitrogen donor [47]. The HisH subunit forms stable dimeric complexes with HisF proteins, constituting IGP synthases. HisF has ammonia-dependent activity that converts the biosynthetic intermediate N1–[(5′-phosphatidyl)methylimino]-5-aminoimidazole-4-carboxamide ribonucleotide into imidazole glycerophosphate and 5-aminoimidazole-4-carboxamide-1-beta-D-ribofuranosyl 5′-monophosphate [48]. In this process, the HisH subunit catalyzes the hydrolysis of glutamine to glutamate and ammonia, with the ammonia being transported to the active site of HisF [48]. The down-regulation of several of the above proteins suggests that VTE and DMY influence normal glutamine metabolism. Additionally, ArgB catalyzes the ATP-dependent phosphorylation of N-acetyl-L-glutamate during arginine biosynthesis [49]. ArgF reversibly catalyzes the transfer of the carbamoyl group from carbamoylphosphate to the N atom of ornithine, producing L-citrulline, the substrate for argininosuccinate synthetase, which catalyzes the final step of arginine biosynthesis [50]. The down-regulation of ArgB and ArgF after exposure to VTE suggests that arginine biosynthesis in *E. coli* is impaired, disrupting normal metabolic processes.

#### 3.5.2. DEPs Associated with Ribosome Assembly and Function

Ribosomes are large biomolecular complexes that play a crucial role in protein synthesis within cells. Our results demonstrate that DMY can down-regulate six distinct ribosomal proteins. An intact ribosome consists of a 30 S small subunit and a 50S large subunit. The large ribosomal protein uL2 (encoded by *rplB*) is crucial for the binding of ribosomal subunits and the binding of tRNAs to the A and P sites [51]. uL22 is a large ribosomal subunit protein with a globular structural domain on the surface and long tentacle-like extensions into the ribosomal core. It forms multiple contacts with various structural domains of the 50S subunit and specifically binds to 23S [52]. Therefore, uL2 and uL22 play essential roles in ribosome function and assembly. Erythromycin has been shown to inhibit the assembly of the 50 S subunit in *E. coli* via L22 [53]. Exposure to DMY resulted in the down-regulation of both uL2 and uL22 proteins, suggesting that DMY affects the assembly and function of large ribosomal subunits. uS3, uS4, uS13, and bS20 are small ribosomal subunit proteins in *E. coli*. uS3 binds to the lower portion of the head of the 30S subunit, interacts with 70S ribosomal mRNA to localize it for translation, and also plays a role in unwinding mRNA during translation [54]. uS4 serves as one of the two initiation proteins for 30S subunit assembly and directly binds to 16S rRNA, where it nucleates the body of the subunit [55]. uS13 is located at the top of the 30S subunit head and interacts with several helices of 16S rRNA. uS13 has C-terminal tails that, along with several features of 16S rRNA, interact with the anticodon stem-loops of P-site tRNAs, potentially playing a functional role in translation [56]. bS20 binds directly to 16S rRNA and is involved in cellular translation and the self-assembly of the small ribosomal subunits. uS13 is located at the top of the 30S subunit head and interacts with several helices of 16S rRNA [57]. Several small ribosomal subunits, including uS3, uS4, uS13, and bS20, were also down-regulated following exposure to DMY. These results suggest that DMY affects the self-assembly and translation functions of both large and small ribosomal subunits, slows down protein synthesis, and interferes with normal cellular activities. Our previous results confirmed that when DMY was applied to *E. coli*, the levels of bacterial intracellular proteins were significantly reduced compared to the control group [15].

### 3.6. DEPs Associated with Nucleotide Metabolism

Disturbances in purine and pyrimidine metabolism in *E. coli* lead to varying degrees of inhibition in energy metabolism and membrane transport. PpnN, pyrimidine nucleotide 5′-monophosphate nucleosidase, catalyzes the hydrolysis of various pyrimidines and purines, degrades excess nucleotides, and partially reintroduces ribose into catabolism to maintain nucleoside pool homeostasis [58]. MazG, a nucleoside triphosphate pyrophosphate hydrolase, hydrolyzes all classical nucleoside triphosphates, which play a role in nutrient regulation for bacterial survival under stress conditions [59]. NrdA can provide precursors necessary for DNA synthesis, catalyzing the conversion of ribonucleotides into deoxyribonucleotides [60]. Exposure to DMY and VTE reduced the expression of PpnN, MazG, and NrdA, suggesting that *E. coli* is impacted by nucleotide synthesis and metabolism, disrupting intracellular homeostasis. CpdA hydrolyzes cAMP to 5′-AMP, which regulates intracellular cAMP concentration, thereby influencing cAMP-dependent processes [61]. Pyrophosphatase (encoded by *rdgB*) hydrolyzes nucleoside triphosphates to their monophosphate derivatives, showing a preference for atypical purine nucleotides such as XTP (xanthine triphosphate), dITP (deoxyinosine triphosphate), and ITP [62,63]. Additionally, the enzyme removes atypical purine nucleotides from the nucleotide pool, preventing their incorporation into DNA/RNA and avoiding chromosomal damage [64]. Hypoxanthine phosphoribosyltransferase (encoded by *hpt*) is a purine-recycling enzyme that transfers the ribulose-5-phosphate moiety from 5-phosphate-alpha-D-ribose 1-bisphosphate to the N9 position of hypoxanthine, producing IMP (inosine 5′-monophosphate) [65]. The down-regulation of CpdA protein, pyrophosphatase, and hypoxanthine phosphoribosyltransferase after VTE exposure suggests that components of VTE, other than DMY, disrupt normal intracellular purine metabolism and destabilize the organism’s internal environment.

### 3.7. Effect of VTE on the Biosynthesis of E. coli Secondary Metabolites

**Figure 5:**
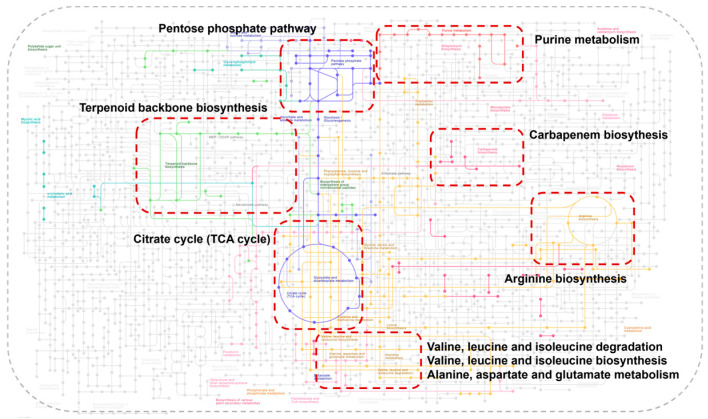
Pathways associated with VTE affecting *E. coli* secondary metabolite biosynthesis process.

Additionally, we discovered that other active compounds in VTE, apart from DMY, could also influence the biosynthesis of secondary metabolites in *E. coli*. This process involved 18 proteins with significant alterations, including 15 being down-regulated and 3 up-regulated. SdaA is a serine dehydratase that catalyzes the deamination of L-serine, resulting in the production of pyruvate and ammonia [66]. Serine hydroxymethyltransferase GlyA catalyzes the reversible interconversion of serine and glycine, using tetrahydrofolate as a one-carbon carrier [67]. This reaction serves as a major source of the one-carbon units required for the biosynthesis of purines, thymidylate, methionine, and other crucial biomolecules. HisC is an aminotransferase that catalyzes the conversion of histidine phosphate and 2-oxoglutarate into L-glutamate and imidazolium acetylphosphate [68]. The chorismate lyase UbiC catalyzes the first step in ubiquinone biosynthesis in *E. coli*, involving the removal of the pyruvoyl group and concomitant cycloaromatization, which converts chorismate into 4-hydroxybenzoate. Phosphoglycolic acid phosphatase GpH specifically catalyzes the dephosphorylation of 2-phosphoglycolate, which is involved in the heterodimerization of the intracellular 2-phosphoglycolate formed during 3′-phosphoglycolic acid DNA repair. It plays a role in salvaging dicarbonyl compounds generated during the cellular DNA repair process [69]. AccA is a subunit of acetyl coenzyme A carboxylase, which catalyzes biotin carboxylation on its carrier protein and the transfer of a CO_2_ group to acetyl coenzyme A, forming malonyl coenzyme A [70]. This reaction represents the first step in bacterial fatty acid biosynthesis. Overall, the active substances in VTE, aside from DMY, can influence *E. coli* secondary metabolite biosynthesis by down-regulating the expression of the aforementioned proteins, thus impacting the normal physiological and metabolic functions of *E. coli*. Figure 5 illustrates how VTE affects the relevant metabolic pathways during the biosynthesis of *E. coli* secondary metabolites.

### 3.8. Protein–Protein Interaction (PPI) Analysis

**Figure 6:**
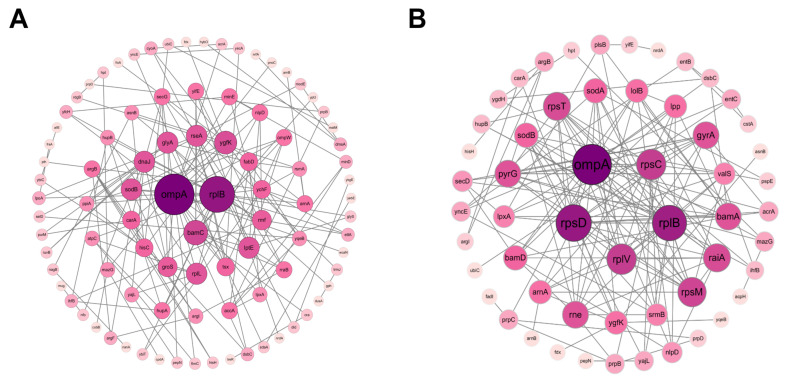
(**A**): Interaction network analysis of differentially expressed proteins between the VTE-treated group and the control group. (**B**): Interaction network analysis of DEPs between the DMY-treated group and the control group. Each node represents a protein and each edge represents an interaction between proteins. Disconnected nodes are hidden.

To investigate the relationship between DEPs in VTE-treated and DMY-treated bacteria, we conducted a visual analysis using a protein–protein interaction (PPI) network. Figure 6 illustrates the protein–protein interaction network of DEPs. Each node represents a protein, while each edge indicates an interaction between proteins. The interaction network of DEPs in the VTE group contains 92 nodes (representing proteins) and 165 edges (representing interactions). The degree values of the outermost circle range from 0 to 3, those of the second outermost circle range from 4 to 5, the middle circle values range from 6 to 9, and the inner circle values range from 10 to 21 (Figure 6A). The PPI network of DEPs in the DMY group contains 55 nodes and 156 edges. The degree values of the outer circle range from 0 to 5, those of the middle circle range from 6 to 12, and the inner circle values range from 13 to 23 (Figure 6B). The color shade in the PPI network indicates the degree of the target: the darker the color, the higher the degree value. Using the Degree algorithm in the CytoHubba plugin, the top five target proteins identified in the VTE group were Outer membrane protein A (OmpA), RplB, BamC, Ygfk, and SodB, while the top five in the DMY group were OmpA, RpsD, RplB, RpsC, and RplV.

Specifically, VTE and DMY down-regulated OmpA 0.75-fold and 0.74-fold, respectively. OmpA is a non-specific transmembrane pore protein that participates in the passive diffusion of various small molecules and plays a key role in maintaining the structural integrity of the bacterial outer membrane [71]. OmpA provides flexible mechanical support for peptidoglycan scaffolds, and its dimerization prevents the local deformation of the peptidoglycan network [72]. OmpA helps *E. coli* resist adversities such as sodium dodecyl sulfate (SDS), bile salts, acidic environments, and high osmolality [71]. The down-regulation of OmpA suggests that VTE and DMY reduce E. coli’s ability to resist external stresses and pathogenicity by modulating OmpA.

### 3.9. Evaluation of Selected Proteins by RT-qPCR

**Figure 7:**
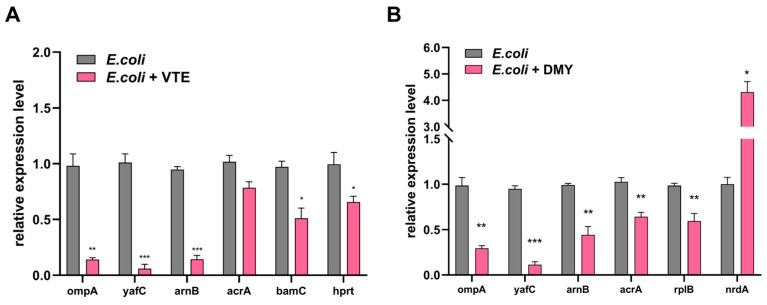
Expression levels of related genes after exposure to VTE and DMY. (**A**) Expression levels of *E. coli*-related genes before and after exposure to DMY. (**B**) Expression levels of *E. coli*-related genes before and after exposure to VTE. Treatment with 0.5 × MIC DMY. We performed three biological replicates.*: *p* < 0.05, **: *p* < 0.01, ***: *p* <0.001.

We conducted RT-qPCR to evaluate the impact of VTE and DMY treatments on the expression levels of *E. coli* genes (Figure 7A,B). The selected genes included outer membrane protein A, HTH-type transcriptional regulator YafC, UDP-4-amino-4-deoxy-L-arabinose-oxoglutarate aminotransferase ArnB, multidrug efflux pump subunit AcrA, large ribosomal subunit protein uL2 (encoded by *rplB*), ribonucleoside-diphosphate reductase 1 subunit alpha NrdA, outer membrane protein assembly factor BamC, and hypoxanthine phosphoribosyltransferase HprT. Among these, the content of YafC exhibited significant changes following DMY and VTE treatments, highlighting the need to further investigate the expression level of the *yafC* gene. YafC is an uncharacterized HTH-type transcriptional regulator from the LysR family, potentially involved in regulating DNA transcription. Previous studies have shown that the plasmid-expressed *yafC* gene enhances the survival of *E. coli* under ionizing radiation [73].

The gene expression levels were consistent with the proteomic results, except for *nrdA*. Ribonucleoside-diphosphate reductase 1 subunit alpha, encoded by *nrdA*, plays a crucial role in *E. coli* nucleic acid metabolism by providing dNTPs for the biosynthesis of deoxyribonucleotides from corresponding ribonucleotides. Exposure to external stimuli affects the nucleic acid metabolism pathway, resulting in DNA denaturation and replication abnormalities. The overexpression of *nrdA* may help bacteria accelerate nucleic acid metabolism and restore normal DNA activity to counteract external environmental stress.

### 3.10. Molecular Docking Analysis of DMY Target Proteins in E. coli

**Figure 8:**
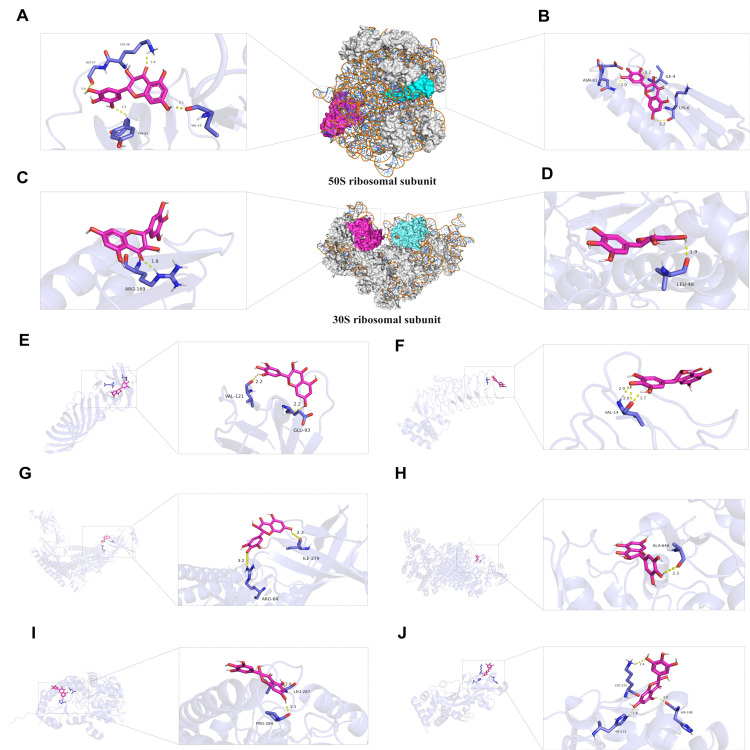
DMY docking modeling of key DEPs: (**A**) DMY and RplB; (**B**) DMY and RplV; (**C**) DMY and RpsC; (**D**) DMY and RpsD; (**E**) DMY and OmpA; (**F**) DMY and LpxA; (**G**) DMY and AcrA; (**H**) DMY and NrdA; (**I**) DMY and ArnB; (**J**) DMY and YafC.

Based on the proteomics results, we identified ten key DEPs as potential targets. Molecular docking was used to evaluate the potential of directly targeting these ten DEPs with DMY. Negative binding energy values suggested a higher likelihood of docking, while larger absolute values indicated more stable interactions between the compound and the protein. Table 1 presents the docking data for DMY with the ten DEPs. The top five protein targets in the PPI rankings of DMY DEPs were first docked (Figure 8A−E). The results revealed that DMY bound more stably to the large ribosomal proteins RplB and RplV, with binding energies of −4.85 kcal/mol and −3.96 kcal/mol, respectively, compared to the small ribosomal subunits RpsC and RpsD, which had binding energies of −3.59 kcal/mol and −3.26 kcal/mol. Furthermore, DMY was docked with five other proteins: AcrA, ArnB, LpxA, YafC, and NrdA. Among these, the binding energy of DMY to YafC was −4.68 kcal/mol, and to LpxA, it was −4.16 kcal/mol, both showing a higher binding stability than the other three proteins. Figure 8 illustrates the docking model of DMY with these proteins.

Molecular docking results suggest that the two large ribosomal subunits of *E. coli*, RplB and RplV; LpxA (involved in lipid A biosynthesis); and YafC (a transcriptional regulator) could be potential repressive binding targets of DMY. These targets primarily contribute to impaired bacterial protein synthesis, the loss of cationic antimicrobial peptide resistance, and disrupted transcriptional regulation.

## 4. Conclusions

**Figure 9:**
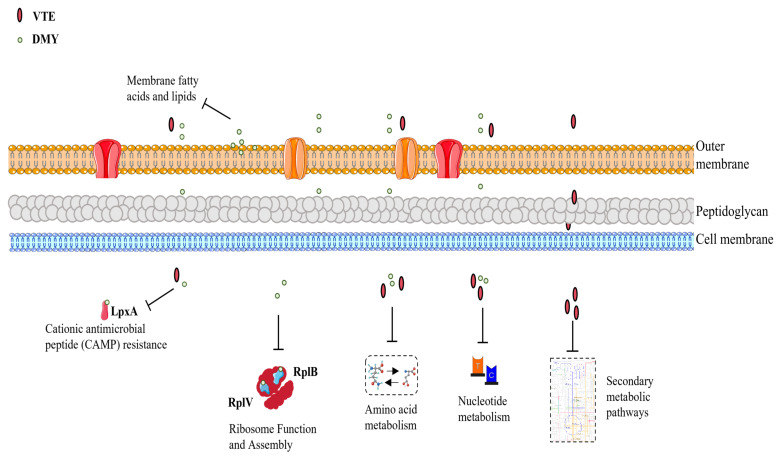
Multi-target mechanism of action of VTE and DMY on *E. coli*.

Previous studies from our group demonstrated that VTE and DMY inhibit *E. coli* by disrupting cell membrane integrity and fluidity, as well as interacting with DNA. However, the effects of VTE and DMY on protein expression, function, and protein–protein interaction in *E. coli* remain unclear. Thus, we further investigated their repressive mechanisms using proteomics, lipid assays, RT-qPCR, and molecular docking. In total, 27 DEPs were up-regulated, and 103 DEPs were down-regulated following VTE exposure in *E. coli*. After exposure to DMY, 15 DEPs were up-regulated, and 66 DEPs were down-regulated in *E. coli*. These DEPs revealed the comprehensive inhibitory mechanisms of VTE and DMY on *E. coli*. As shown in Figure 9, VTE and DMY inhibit *E. coli* growth by disrupting the cationic antimicrobial peptide resistance pathway, amino acid biosynthesis and metabolism, and nucleotide metabolism. Additionally, VTE disrupts various secondary metabolic pathways, while DMY interferes with E. coli ribosome assembly and function, and disrupts cell membrane lipid homeostasis by interfering with fatty acid metabolism. RT-qPCR was used to further validate the gene expression levels of key proteins. The molecular docking results indicate that RplB, RplV, LpxA, and YafC are likely key targets of DMY in *E. coli*. These findings offer new insights into the multi-target inhibition mechanism of VTE and DMY against *E. coli*. Our further studies will investigate the impact of DMY on these proteins using Western blotting or parallel reaction monitoring.

## Figures and Tables

**Table 1:** Molecular docking analysis of 10 key proteins of *E.coli* by DMY.

*E. coli* Key Protein Targets	Binding Energy (kcal/mol)	Number of Hydrogen Bonds	Hydrogen Bonding Residues of the Enzyme
OmpA	–2.99	2	VAL-121, GLU-93
RplB	–4.85	4	TYR-83,GLY-27;VAL-19;LYS-26
RplV	–3.96	3	ILE-4;ASN-6;LYS-6
RpsD	–3.26	1	LEU-48
RpsC	–3.59	1	AGR-169
AcrA	–2.21	2	ARG-64; ILE-279
ArnB	–2.91	2	PRO-284; LEU-287
LpxA	–4.16	3	VAL-14
YafC	–4.68	3	HIS-188, HIS-212, LYS-215
NrdA	–1.91	1	ALA-646

## Data Availability

The original contributions presented in this study are included in the article/Supplementary Materials. Further inquiries can be directed to the corresponding authors.

## Supplementary Materials

The following supporting information can be downloaded at: https://www.mdpi.com/article/10.3390/foods14122011/s1, Table S1: Differential protein information after VTE treatment of *E. coli*; Table S2: Differential protein information after DMY treatment of *E. coli*.

## Appendix A

**Table A1:** Operating parameters of mass spectrometers.

Parameters	Numerical Setting
Mass range	50–1200 M/Z
Capillary voltage	3200 V(+)/2500 V(−)
Resolution	>40,000 FWHM
Ionization mode	ESI dual mode switching
Sampling cone voltage	35 V
Desolvation temperature	400 °C
Desolvation gas flow	800 L/H
Cone gas flow	50 L/H
Source temperature	120 °C

**Table A2:** Information about each primer in PCR experiments.

Target Gene	Primer	Primer Sequence (5′−3′)
16 S rRNA	16 S rRNA-F	GCTGCCCTTTGTATTGTC
	16 S rRNA-R	AGATGTTGGGTTAAGTCCC
ompA	ompA-F	TCCAGAGCAGCCTGACCTTC
	ompA-R	GCTGAGCCTGGGTGTTTCCT
yafC	yafC-F	AGTGAATTCAAGGAGATATACCA
	yafC-R	AGTAAGCTTTTAAGCCTCTCT
rpsC	rpsC-F	AGGAACTGGCTAAAGCGTCCG
	rpsC-R	CGACCTTCGCGGTACCATTCG
arnB	arnB-F	GCCTTCCCTGACCTGGGTTTC
	arnB-R	ATACGTACCGACGGCATGGGC
acrA	acrA-F	GAGTACGATCAGGCTCTGGCTG
	acrA-R	GCGTGCCATTCGCCAGTTCC
bamC	bamc-F	TGCTGGTGGAAAATGGTCGTGG
	bamc-R	TCCGTGCTGTAACGCTGCATG
hpt	hpt-F	CGACTTTATGACCGCCTCCAGC
	hpt-R	GGCAGATGACGGTAACGCTGTG
nrdA	nrdA-F	CCAGACAGCGGTGAAATCCTG
	nrdA-R	TTCCTGATCGGCGAAGAACGC
